# Nutrient Effects on Motor Neurons and the Risk of Amyotrophic Lateral Sclerosis

**DOI:** 10.3390/nu13113804

**Published:** 2021-10-26

**Authors:** Polina S. Goncharova, Tatiana K. Davydova, Tatiana E. Popova, Maxim A. Novitsky, Marina M. Petrova, Oksana A. Gavrilyuk, Mustafa Al-Zamil, Natalia G. Zhukova, Regina F. Nasyrova, Natalia A. Shnayder

**Affiliations:** 1 Center of Personalized Psychiatry and Neurology, V.M. Bekhterev National Medical Research Centre for Psychiatry and Neurology, 192019 Saint-Petersburg, Russia; po.gon4arova@yandex.ru (P.S.G.); maximnovitsky93@gmail.com (M.A.N.); 2 Center of Neurogenerative Disorders, Yakut Science Centre of Complex Medical Problems, 677000 Yakutsk, Russia; tanya.davydova.56@inbox.ru (T.K.D.); tata2504@yandex.ru (T.E.P.); 3 Center for Collective Using “Molecular and Cell Technologies”, V.F. Voino-Yasenetsky Krasnoyarsk State Medical University, 660022 Krasnoyarsk, Russia; stk99@yandex.ru (M.M.P.); oksana.gavrilyuk@mail.ru (O.A.G.); 4 Department of Physiotherapy, Faculty of Continuing Medical Education, Peoples’ Friendship University of Russia, 117198 Moscow, Russia; alzamil@mail.ru; 5 Department of Neurology and Neurosurgery, Siberian State Medical University, 634050 Tomsk, Russia; znatali@yandex.ru

**Keywords:** amyotrophic lateral sclerosis, predictor, risk factor, protector, nutrient, vitamin

## Abstract

Amyotrophic lateral sclerosis (ALS) is an incurable chronic progressive neurodegenerative disease with the progressive degeneration of motor neurons in the motor cortex and lower motor neurons in the spinal cord and the brain stem. The etiology and pathogenesis of ALS are being actively studied, but there is still no single concept. The study of ALS risk factors can help to understand the mechanism of this disease development and, possibly, slow down the rate of its progression in patients and also reduce the risk of its development in people with a predisposition toward familial ALS. The interest of researchers and clinicians in the protective role of nutrients in the development of ALS has been increasing in recent years. However, the role of some of them is not well-understood or disputed. The objective of this review is to analyze studies on the role of nutrients as environmental factors affecting the risk of developing ALS and the rate of motor neuron degeneration progression. Methods: We searched the PubMed, Springer, Clinical keys, Google Scholar, and E-Library databases for publications using keywords and their combinations. We analyzed all the available studies published in 2010–2020. Discussion: We analyzed 39 studies, including randomized clinical trials, clinical cases, and meta-analyses, involving ALS patients and studies on animal models of ALS. This review demonstrated that the following vitamins are the most significant protectors of ALS development: vitamin B12, vitamin E > vitamin C > vitamin B1, vitamin B9 > vitamin D > vitamin B2, vitamin B6 > vitamin A, and vitamin B7. In addition, this review indicates that the role of foods with a high content of cholesterol, polyunsaturated fatty acids, urates, and purines plays a big part in ALS development. Conclusion: The inclusion of vitamins and a ketogenic diet in disease-modifying ALS therapy can reduce the progression rate of motor neuron degeneration and slow the rate of disease progression, but the approach to nutrient selection must be personalized. The roles of vitamins C, D, and B7 as ALS protectors need further study.

## 1. Introduction

Amyotrophic lateral sclerosis (ALS) is an incurable chronic progressive neurodegenerative disease with the progressive degeneration of motor neurons in the motor cortex and lower motor neurons in the spinal cord and the brain stem [1]. As a rule, patients die from respiratory failure 3–5 years after onset of the disease. The incidence rate of ALS worldwide varies from 0.4 per 100,000 a year to 3.83 per 100,000 a year and its prevalence-from 0.1 per 100,000 population to 42.1 per 100,000 population [2]. The etiology and pathogenesis of ALS remain not fully understood. Therefore, there is still no single concept explaining the nature of ALS [3]. A study of the risk factors for ALS will help us to understand the development mechanisms of this disease and, possibly, to develop a specific etiotropic and disease-modifying therapy. In recent years, the genetic and environmental predictors of ALS have been actively studied [4]. Specifically, the protective/predictive role of nutrients in the development of ALS and in slowing the rate of ALS–associated motor neurons degeneration has recently gained increasing research interest, which has prompted us to prepare this review.

## 2. Materials and Methods

We searched for full-text English and Russian language publications in the PubMed, Springer, Clinical keys, Google Scholar, and E-Library databases using the following keywords and their combinations: amyotrophic lateral sclerosis—ALS, predictor, risk factor, protector, nutrient, and vitamin. We analyzed all the available studies published in 2010–2020. In total, 672 publications were analyzed, 39 of which were review studies (including randomized clinical trials, clinical cases, and meta-analyses with the participation of ALS patients, as well as studies with the use of an animal model of ALS) consistent with the purpose of the present one. In addition, studies of historical interest were included in the review. Despite an extensive search, it is possible that some publications were not taken into consideration.

## 3. Results

Nutrition is the study of nutrients in food; how the body uses them; and the relationships between diet, health, and disease. Nutrition also focuses on how people can use dietary choices to reduce the risk of disease. Nutrients provide nourishment. Proteins, carbohydrates, fat, vitamins, minerals, fiber, and water are all nutrients. If people do not have the right balance of nutrients in their diet, their risk of developing certain health conditions increases [5]. Vitamins are organic substances present in minute amounts in natural foodstuffs. Having too little of any particular vitamin may increase the risk of developing certain health issues. A vitamin is an organic compound, which means that it contains carbon. It is also an essential nutrient that the body may need to get from food. Vitamins as organic nutrients play an important role in the development and functioning of the central nervous system (CNS). ALS patients’ awareness of different vitamins and their effects can help these patients to make sure they get enough of the vitamins they need [6]. Much research has been devoted to investigating the role of vitamins in the etiology of ALS. However, there is no single approach to their use in ALS. In this article, we consider the function of vitamins and other nutrients in the development and progression of ALS. We analyze both the well-known and promising nutrients capable of reducing the risk and rate of motor neuron degeneration in patients with ALS.

### 3.1. Vitamin A

Vitamin A (carotenoids and trans-retinol and its esters) is a group of fat-soluble substances that enter the body with food in the form of retinol esters and carotenoids, which are then metabolized into active compounds and are involved in many physiological processes, such as the neutralization of free radicals, regulation of protein synthesis, regulation of cell growth, and differentiation [7,8] (Table 1). Due to the variety of functions vitamin A performs, a lot of research has been devoted to its role in recent years, including the study of the relationship between the serum vitamin A level and the risk of ALS.

We found two studies of the role of vitamin A in the development of ALS. One study confirmed the protective role of vitamin A in the development of ALS [9], while another study showed questionable results [10].

Specifically, Fitzgerald et al. (2013) analyzed the risk of ALS associated with the carotenoid intake in five prospective cohort studies including 96,937 males and 118,843 females (45–75 years old). Cox proportional hazards regression was applied to calculate the risk ratios for specific cohorts. As a result, the authors concluded that the increased total consumption of major carotenoids had a protective effect and reduced the risk of ALS [9]. One limitation of the study was that ALS death rather than incidence was used in CPS-II Nutrition (the Cancer Prevention Study II-Nutrition Cohort), MEC (the Multiethnic Cohort Study), and NIH-AARP (the National Institutes of Health–Association of American Retired Persons Diet and Health Study). However, due to the rapidly progressive nature of ALS (median survival ¼ 1.5–3 years), the authors assumed ALS death to be a reasonable proxy for incidence.

**Figure 1:**
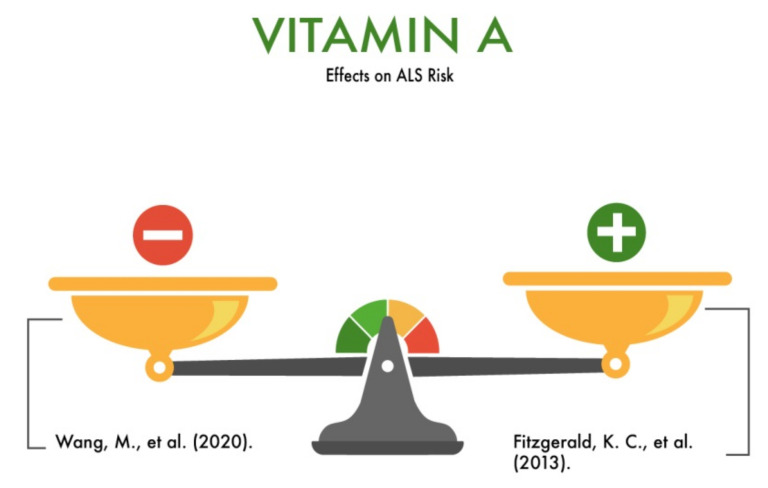
The effect of vitamin A on the risk of developing amyotrophic lateral sclerosis (ALS).

Wang et al. (2020) [10] studied the level of vitamin A in the blood serum of healthy people and 202 patients with ALS. In addition to vitamin A, the authors also took into account the levels of vitamins E, B2, B9, and C, as well as the cholesterol level. According to the results obtained, higher levels of vitamins A and E and lower levels of vitamins B2, B9, and C were found in patients with ALS compared with those in the control group. Accordingly, vitamin B2, B9, or C deficiency/hypovitaminosis can be considered as a risk factor or ALS predictor. Additionally, in the present study, the serum vitamin A level was significantly higher in patients with ALS compared to the healthy controls. In addition, the OR of vitamin A for ALS was significantly >1, indicating that high levels of vitamin A were associated with an increased risk of ALS. However, the pooled results from five cohort studies showed that a high intake of carotenoids (a main source of vitamin A) was associated with a reduced risk of ALS. Concurrently, according to these study results, the effects of the vitamin A and E levels on the ALS risk are questionable. In view of this, the role of vitamin A in the development of ALS needs clarification and additional research (Figure 1).

### 3.2. Vitamin B1

Vitamin B1 (aneurin and thiamine) is a water-soluble vitamin that is synthesized by plants and some microorganisms. Vitamin B1 comes from food in a free in an esterified and partially bound form. Under the influence of digestive enzymes, it is converted into free thiamine, which is absorbed from the small intestine. Most of the thiamine that enters the blood is quickly phosphorylated in the liver, a smaller part of it enters the blood in the form of free thiamine and is redistributed to other tissues, and a part of it is again excreted into the gastrointestinal tract, along with bile and excretions of the digestive glands. Thus, a constant recirculation of vitamin B1 and a gradual, uniform assimilation of it by tissues is ensured [11]. The kidneys actively excrete vitamin B1 into the urine. An adult excretes from 100 to 600 μg of thiamine per day. The introduction of increased amounts of vitamin B1 with food parenterally increases its excretion in the urine, but as the doses increase, the proportionality gradually disappears. In human urine, along with thiamine, its decay products begin to appear in increasing quantities, which, with the intake of vitamin B1 over 10 mg per day, can amount to 40–50% of the initial dose. Almost half of vitamin B1 can be found in muscles, 40% of it can be found in internal organs, and 15–20% in the liver [12]. Vitamin B1 plays an important role in the functioning of the CNS and the PNS [13] (Table 1).

Jesse et al. (2015) conducted a study in Finland involving 122 ALS patients (median age 62 ± 13 years, 62/60 males/females; 22 patients had bulbar, 29 spinal, and 71 spinal and bulbar symptoms (stated at the time of involvement). The patients’ blood thiamine levels were checked based on the erythrocyte transketolase (TK) activity and the TK activity after the addition of thiamine pyrophosphate (TPP effect). In the case of thiamine deficiency, the basal MC activity increased after the addition of TPP. Pathological TPP effects were found in 28% of the cases. Among these patients, 41% had a mild vitamin B1 deficiency, while 59% had a severe deficiency of this vitamin [14]. Additionally, the authors showed that the clinical triad of Wernicke encephalopathy (mental impairment, ophthalmoplegia, and gait ataxia) in patients with ALS can be found in only ∼10% of cases, but a diagnosis is made before autopsy in merely 15% of cases. However, no clinical diagnostic procedure (MRI or TPP effect) can confirm the sole diagnosis of Wernicke encephalopathy in ALS patients with a vitamin B1 deficiency.

Liu et al. (2016) conducted a meta-analysis of studies examining the role of vitamin B1 deficiency in the development of various neurodegenerative diseases, including ALS. The authors demonstrated that TPP, the main active form of thiamine, functions as an essential cofactor for a number of thiamine-dependent enzymes, such as transketolase, pyruvate dehydrogenase complex (PDHC), and KGDHC. Once the levels of TTP are diminished in the human brain, the activity of these enzymes is affected, leading to alterations in the mitochondrial activity, impairment of oxidative metabolism, decreased energy status, and brain damage. This process may be important for ALS development. In the course of the study, the authors came to the conclusion that a vitamin B1 deficiency causes oxidative stress and a disturbance in the concentration of intracellular calcium, which may be one of the reasons for the development of ALS and other neurodegenerative diseases [15].

**Figure 2:**
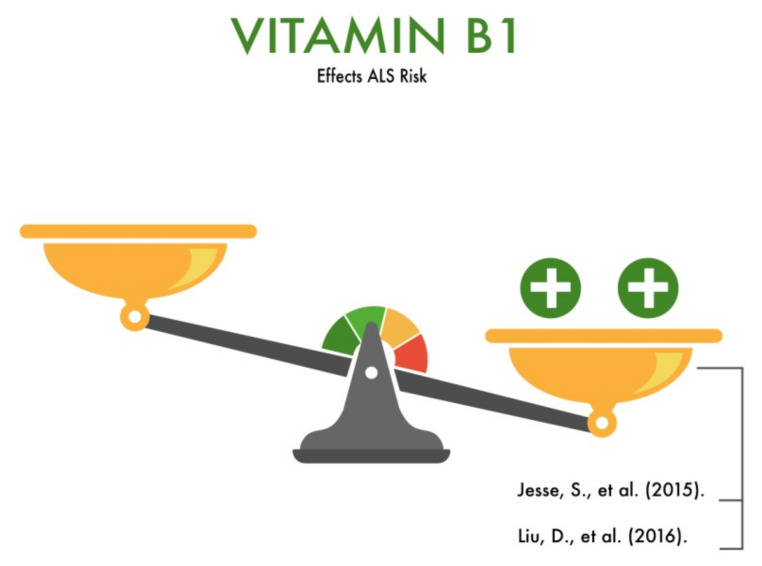
The effect of vitamin B1 on the risk of developing amyotrophic lateral sclerosis (ALS).

Thus, despite a small number of studies, vitamin B1 can be considered as a protector for the development of ALS (Figure 2). However, the number of clinical observations and randomized trials should be increased in the future.

### 3.3. Vitamin B2

Vitamin B2 (riboflavin) is a water-soluble vitamin that enters the human body along with food, but it can be synthesized in small amounts by intestinal bacteria [16,17]. Vitamin B2 is involved in many biological processes in the CNS and PNS (Table 1). It is absorbed best in the presence of hydrochloric acid in gastric juice; however, it is absorbed in the small intestine as well. After its absorption in the intestine, vitamin B2 undergoes phosphorylation [18]. The absorption chemically bound to a protein of vitamin B2 occurs after it is released from the protein during phosphorylation. This produces two coenzyme forms of vitamin B2: flavin mononucleotide (FMN) and flavin adenine dinucleotide (FAD). The phosphorylation of riboflavin can occur not only in the intestinal mucosa but also in the cells of the liver, blood, and other cells of the body as well. With vitamin B2 deficiency, the excretion of riboflavin in the urine approaches zero [12].

Due to the wide range of functions of vitamin B2 in the CNS and PNS (Table 1), a lot of studies are devoted to the investigation of the role of this vitamin in the mechanisms of neurodegenerative disease development. However, currently, there is a lack of research devoted to ALS.

**Figure 3:**
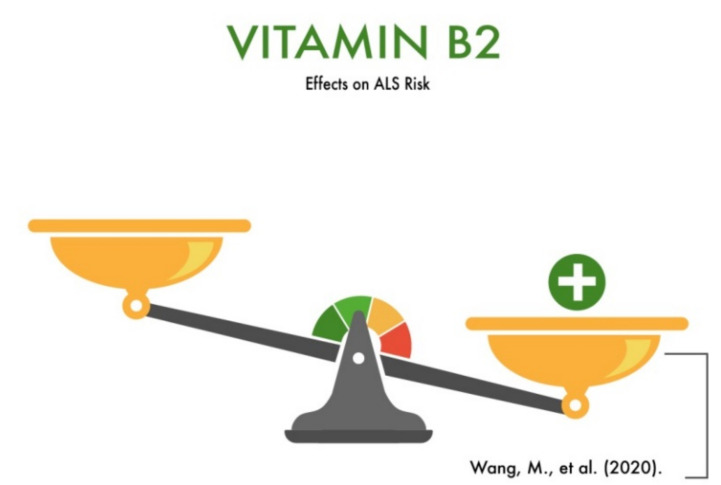
The effect of vitamin B2 on the risk of developing amyotrophic lateral sclerosis (ALS).

Wang et al. (2020) conducted a study involving 202 ALS patients and 208 healthy subjects in China. All study participants were from the Hunan Province of China and had similar dietary habits. Venous blood samples were taken from all of them to study the nutrient levels, including vitamin B2. The results showed that the levels of vitamins (A, B2, B9, C, and E) in ALS patients differed significantly from those of the healthy study participants. Specifically, a lower level of vitamin B2 was found in patients with ALS compared to healthy people [10]. In addition, vitamin B2 is a precursor of the coenzymes flavin mononucleotide (FMN) and flavin adenine dinucleotide (FAD), which are essential in the mitochondrial electron transport chain and responsible for regenerating the antioxidant glutathione reductase. Thus, vitamin B2 acts indirectly as an antioxidant. Neurological abnormalities, such as ataxia and an inability to stand due to riboflavin deficiency, have been reported. This made the authors hypothesize that vitamin B2 deficiency is a predictor of ALS development (Figure 3). It should be recognized that further research is needed on the protective role of vitamin B2 in the degeneration of motor neurons and the development of ALS in other ethnic groups.

### 3.4. Vitamin B6

Vitamin B6 (adermine and pyridoxine) is a water-soluble vitamin that enters the human body with food. However, it is also synthesized by the intestinal microflora. It is difficult to determine the daily requirement for vitamin B6 in humans, since a significant amount of it is synthesized by the intestinal microbiome [19].

Vitamin B6 plays a key role in the development and regulation of the CNS and PNS functions [13] (Table 1) in humans, as well as in the development of autoimmune and inflammatory diseases [20,21]. That is why foodstuffs and medicines containing pyridoxine are actively used in neurology and psychiatry.

The availability of methods for the differential determination of the serum level of vitamin B6 made it possible to more thoroughly investigate the exchange of its various forms [22]. It is rapidly absorbed from the intestines and undergoes further transformations in the tissues of the body. However, the transformations of vitamin B6 in the human body are still under investigation.

Introduced vitamin B6 is excreted in the urine in the form of its metabolites (pyridoxylic acid and its lactone; the Schiff’s base of pyridoxal) 2–4 h after administration. At the same time, only insignificant amounts of free pyridoxine and other forms of the vitamin were found in the urine. As already mentioned, the final metabolic product of all forms of vitamin B6 is 4-pyridoxylic acid, the excretion of which in the urine (in total with lactone) is 70–90% of the introduced vitamin B6. The exchange of vitamin B6 in the body is closely related to the processes of biosynthesis and dissimilation of pyridoxal phosphate, the main coenzyme form of vitamin B6, which is part of various enzyme systems. However, pyridoxine itself is not active as a metabolic cofactor and should be considered a provitamin. Through a series of enzymatic reactions, pyridoxine is converted in the body into biologically active forms of vitamin B6–pyridoxal phosphate and pyridoxamine phosphate [13,22]. After the administration of pyridoxamine, in addition to pyridoxal acid and unchanged amine, pyridoxal is excreted in the urine. In people exposed to high temperatures and humidity, various metabolites of vitamin B6, in addition to urine, are found in sweat [12].

**Figure 4:**
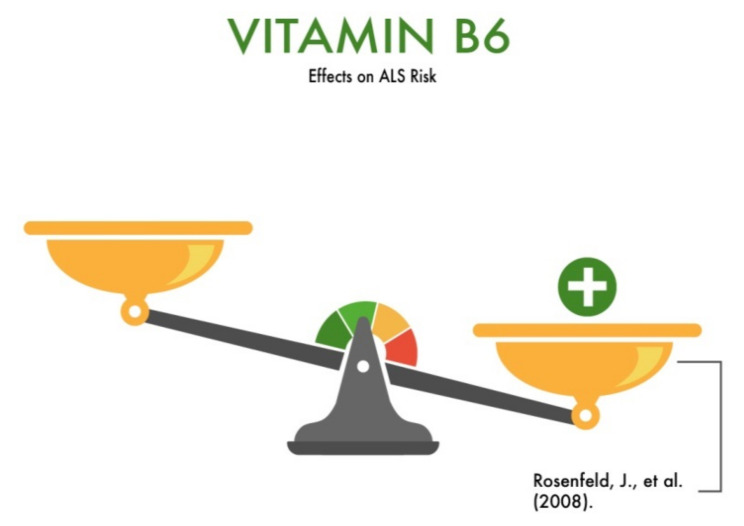
The effect of vitamin B6 on the risk of developing amyotrophic lateral sclerosis (ALS).

Rosenfeld et al. (2008) [23] published a large meta-analysis that covered the role of nutrients, including vitamin B6, in maintaining survival and treating ALS patients. The authors concluded that a vitamin B6 deficiency may play a significant role in the etiology of ALS. Additionally, this article considers elevated plasma homocysteine levels in ALS patients [24] as a risk factor for ALS. In turn, vitamin B6 converts homocysteine into sulfuric amino acids [25], which may play a protective role in this neurodegenerative disease (Figure 4). Thus, taking into account the role of vitamin B6 in the development and regulation of the central nervous system and metabolism, one can recognize its protective role in the development and progression of ALS. However, the study of the protective role of this vitamin in the degeneration of motor neurons needs to be continued.

### 3.5. Vitamin B7

Vitamin B7 (B8, H, and biotin) is a water-soluble vitamin that enters the human body mainly with food. Little is known about biotin metabolism in neurodegenerative diseases, but research continues [26,27]. Biotin, taken from food in a bound state, is cleaved from the protein by the action of proteolytic enzymes, turns into a water-soluble form, and is absorbed into the bloodstream in the small intestine. The intestine also absorbs biotin synthesized by bacteria in the gastrointestinal tract. The biotin absorbed into the blood binds to serum albumin and is carried throughout the body. The greatest amount of biotin accumulates in the liver, kidneys, and adrenal glands and in the brain in men, where biotin accumulates more than twice (0.08 μg/g) the amount than in women (0.03 μg/g), which probably should be taken into account when planning the choice of the dose of food and medicinal products containing biotin for men and women suffering from neurodegenerative diseases and children with neurodevelopmental disorders [12].

Vitamin B7 is involved in such physiological processes as the regulation of energy metabolism, carboxylation reactions, synthesis of purines, fatty acid metabolism, regulation of the circadian cycle, and much more [28,29] (Table 1). This indicates its important role in the prevention and treatment of neurological diseases and mental disorders [30].

Mangelsdorf et al. (2017) published a clinical case in which a special individual diet was developed for a 49-year-old patient with ALS, including a daily intake of biotin at a dose of 5 mg per day. Along with biotin, the diet also included retinol, thiamine, riboflavin, niacinamide, adermine, folic acid, cyanocobalamin, ascorbic acid, cholecalciferol, alphatocopherol, and a mineral complex. ALS therapy also included daily exercise, the etiotropic treatment of concomitant diseases, and regular monitoring of the laboratory and instrumental parameters. The authors described the results over three years of treatment. As a result of three years of individually developed therapy, the patient’s general well-being improved; the patient started jogging and went to the gym regularly. He gained muscle and body mass. His athletic performance was even better than before being diagnosed with ALS [31].

**Figure 5:**
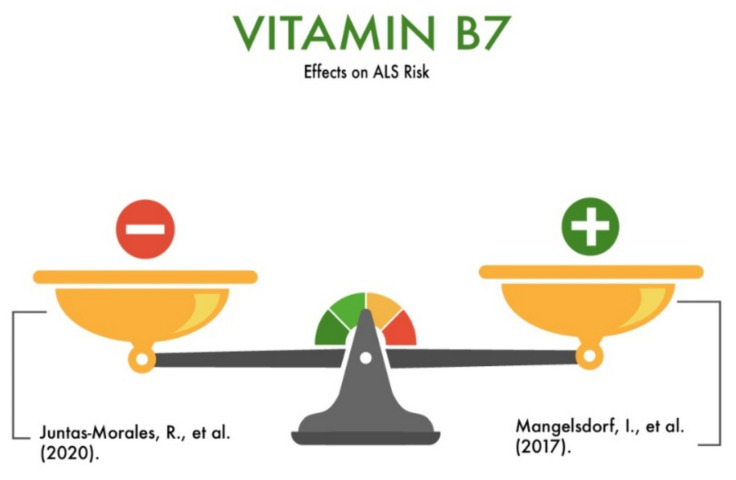
The effect of vitamin B7 on the risk of developing amyotrophic lateral sclerosis (ALS).

Juntas-Morales et al. (2020) conducted a study involving 30 ALS patients. The study examined the effect of MD1003 (high-dose pharmaceutical grade biotin) on the course of ALS. The drug was safe and well-tolerated, but the effectiveness of biotin as a disease-modifying therapy for ALS remained unproven [32]. Thus, the role of vitamin B7 in the development of ALS is significant but needs to be clarified (Figure 5).

### 3.6. Vitamin B9

Vitamin B9 (folic acid and folate) is a water-soluble vitamin that enters the body with food [33] and can be produced by the intestinal microbiome [34,35]. The absorption of folic acid is carried out mainly in the duodenum and the proximal part of the small intestine. In the ileum and cecum, folate is almost not absorbed. Of particular interest is the question of the absorption of various dietary folates. Due to the fact that folates are contained in food products, mainly in a reduced form of polyglutamates and their formyl and methyl derivatives, the possibility of absorption of the latter is a subject of special study. Most monoglutamates are readily absorbed, while polyglutamates are absorbed only after excess glutamic acid is removed by intestinal enzymes-gamma-glutamyl carboxypeptidases or conjugates. Therefore, a number of experts from the World Health Organization (WHO) consider it appropriate to judge the content of folic acid in food or a diet only by the amount of “free” folates and not take into account the presence of polyglutamates containing more than three residues of glutamic acid.

In the intestine, folic acid is reduced by the enzyme dihydrofolate reductase to tetrahydrofolic acid (THPA), then methylated. A violation of the restoration of folate in the intestine in some diseases leads to impaired absorption of this vitamin. The absorbed folates enter the liver, where they accumulate and are converted into the active forms. The body of an adult contains about 7–12 mg of folates, of which about 50–70% (5–7 mg) is in the liver. The ability of the liver to accumulate and use folates is in direct proportion to the supply of the body with certain nutritional factors: proteins, individual amino acids, and vitamins. About 60% of serum folate is bound to serum proteins. Serum folates are mostly unconjugated and represented by N-methyl-THPA.

Despite the fact that the main amount of blood folates is contained in erythrocytes, more often for diagnostic and other purposes, the determination of the level of folates in the serum is used. According to a number of authors, the content of folate in the blood serum of a healthy person ranges from 6 to 20 ng/mL [12]. Folic acid performs a number of important functions in the human body, such as participation in the formation of erythrocytes and leukocytes, participation in the processes of iron metabolism, participation in the synthesis of DNA nucleotides, and other processes (Table 1) [36,37].

The biochemical functions of folates are closely related to the metabolism of proteins and amino acids. As indicated above, by including folic coenzymes in the exchange of one-carbon compounds, they participate in the biosynthesis of such important precursors of nucleic acids as purine and pyrimidine bases.

Additionally, folates are involved in the exchange of a number of amino acids, including serine, glycine, histidine, methionine, tryptophan, and others. In turn, the conversion of folates into their active forms largely depends on the state of the body’s protein supply. Protein starvation interferes with both the liver’s ability to convert pteroylglutamic acid into its active form and the organ’s ability to store it. Therefore, the role of folic acid is very important in the prenatal period [38].

Wang et al. (2020) conducted a study involving ALS patients and healthy people. The volunteers from the Hunan Province of China had similar dietary habits. The study found that the serum folate levels were lower in ALS patients than in controls. The authors suggested that this may be due to the fact that folic acid can indirectly reduce the risk of multifactorial neurodegenerative diseases by reducing the level of homocysteine in the blood [39]. The authors also suggested that folic acid can be used as a prophylaxis and treatment for ALS, since supplementation with folic acid made it possible to delay the onset of the disease and prolong the life expectancy in transgenic ALS mice by decreasing the plasma homocysteine levels [10,40]. The levels of tetrahydrofolate decreased significantly at the middle-to-late stages of the disease in a mouse model of ALS. Thus, low serum vitamin B9 may increase the risk of ALS by affecting the homocysteine levels.

**Figure 6:**
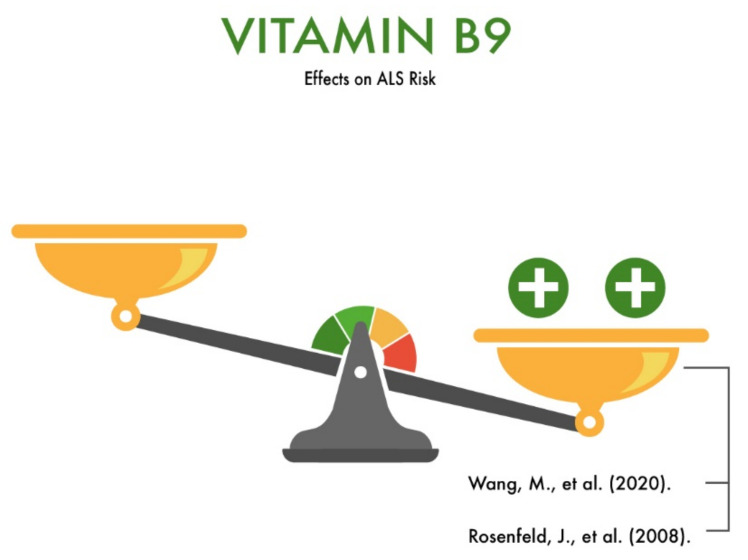
The effect of vitamin B9 on the risk of developing amyotrophic lateral sclerosis (ALS).

Rosenfeld et al. (2008) published a large meta-analysis looking at the role of nutrients, including folate, in the maintenance and management of ALS patients. The authors concluded that a folate deficiency may play a significant role in the etiology of ALS. Elevated plasma homocysteine levels due to impaired folic acid metabolism in people with ALS was considered a risk factor for ALS [24]. The authors concluded that the intake of vitamin B, primarily folates and methylcobalamin, can reduce the plasma homocysteine levels [24] and, thus, reduce the risk of developing ALS and the rate of its progression [23] (Figure 6).

Therefore, the protective role of folate in reducing the risk and slowing the rate of progression of motor neuronal degeneration can be considered proven. However, it is important to consider the content of active folates in foods and vitamin and mineral complexes.

### 3.7. Vitamin B12

Vitamin B12 (cyanocobalamin) is a water-soluble vitamin that enters the human body with food and can be synthesized by lactobacilli [37]. Cyanocobalamin is the only vitamin synthesized exclusively by microorganisms [41]; neither plants nor animal tissues are endowed with this feature. In humans and animals, a vitamin B12 deficiency leads to the development of malignant macrocytic, megaloblastic anemia [42], and increases the risk of congenital malformations.

In addition to changes in hematopoietic functions, disorders in the activity of the CNS and PNS and a sharp decrease in the acidity of gastric juice and the content of a special protein, gastromucoprotein, called the internal Castle factor, which specifically binds cyanocobalamin into a special complex, are also specific for vitamin B12 deficiency. The exact role of this factor in vitamin absorption has not been elucidated. It is assumed that, in the complex associated with the internal Castle factor, cyanocobalamin enters the cells of the mucous membrane of the ileum and then slowly passes into the blood of the portal system. In this case, the internal Castle factor undergoes hydrolysis (decomposition) [12].

Vitamin B12 performs a number of important functions in the body [36,37]: regulation of the folate cycle, participation in the regulation of the amino acids and fatty acids, participation in the regulation of the growth and differentiation of neurons, participation in the regulation of the formation of the myelin sheath, and others (Table 1).

It should be noted that cyanocobalamin enters the blood of the portal system not in a free state but in a complex with two proteins called transcobalamins I and II, one of which serves as a B12 (I) depot, since it binds more strongly to vitamin B12. Therefore, a violation of the synthesis of Castle’s internal factor in the gastric mucosa leads to the development of a vitamin B12 deficiency, even if there is a sufficient amount of this vitamin in the food. In such cases, vitamin B12 for therapeutic purposes is usually administered parenterally or with food but, also, in combination with neutralized gastric juice, which contains an intrinsic Castle factor.

In a meta-analysis, Rosenfeld et al. (2008) [23] reviewed the roles of nutrients, including vitamin B12, in improving the survival of ALS patients. The authors analyzed a study in which high doses of methylcobalamin (an analog of cyanocobalamin) were used as maintenance therapy in patients with ALS, including its effect on the average amplitudes of the action potential of the skeletal muscle. There were two groups in the study: the low-dose group and the high-dose methylcobalamin group. In the group of low doses of methylcobalamin, no significant changes were found in the amplitude of the action potential in comparison with the baseline level. However, eight patients in the high-dose group showed significantly higher values of the amplitude of the action potential (*p* < 0.05). According to the authors, high doses of methylcobalamin can enhance the work of motor neurons [43]. The results obtained indicate that high doses of methylcobalamin are promising for the development of new strategies for disease-modifying ALS therapy. However, prospective placebo-controlled studies are required to evaluate the efficacy and safety of high doses of methylcobalamin in ALS patients.

Rison and Beydoun (2010) published a clinical case and a literature review showing the prognostically negative role of vitamin B12 deficiency on the progression of ALS [44]. According to the authors, laboratory studies for ALS patients should include studies of the serum vitamin B12 levels, as this vitamin plays a role in the combined degeneration of motor neurons along with monoclonal gammopathy. A B12 deficiency is associated with megaloblastic anemia, glossitis, dementia, peripheral neuropathy, and myelopathy. In particular, deficiencies of vitamin B12 may cause the subacute combined degeneration of the spinal cord. This disorder shares upper motor neuron signs with ALS, and hence, it is customary to measure the B12 levels in the investigation of not only ALS but, also, all peripheral neuropathies because of the readily available treatments for deficiency. Fortunately, B12-deficient neuropathies have different characteristics from that of MND/ALS [44].

Moreover, Zhang et al. (2010) showed that the inclusion of vitamin B12 in disease-modifying therapy for ALS statistically significantly affects the timing of the onset of the disease, improves the quality and duration of life in ALS in humans and in an animal model (*SOD1* G93A transgenic mice) due to a decrease in the level of homocysteine in the plasma, suppression of microglial and astrocytic activation, inhibition of inducible oxidase synthase, and expression of tumor necrosis factor-alpha [45].

Japanese researchers (2015) showed that the intraperitoneal administration of ultra-high doses of methylcobalamin to transgenic mice with ALS inhibits the progression of the disease [46].

**Figure 7:**
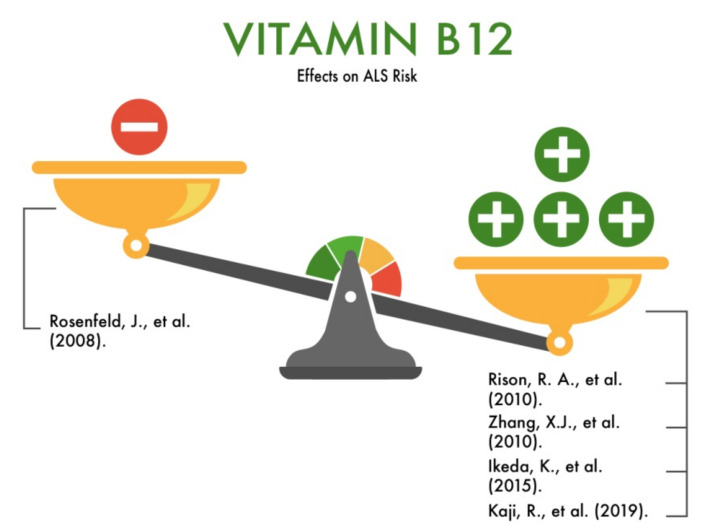
The effect of vitamin B12 on the risk of developing amyotrophic lateral sclerosis (ALS).

Kaji et al. (2019), as the result of an extended second/third phase of a randomized controlled trial involving patients with ALS, showed that ultra-high doses of methylcobalamin slow down the severity of the symptomatic progression of the disease and increase the life expectancy of patients with ALS if methylcobalamin is prescribed early [47]. Thus, vitamin B12 may have a protective effect on the risk of developing ALS (Figure 7) and is recommended for the treatment of this disease.

### 3.8. Vitamin C

Vitamin C (ascorbic acid and ascorbates) is a water-soluble vitamin that enters the human body with food. Ascorbic acid is one of the most important nutrients in the human diet, as it is involved in many processes, including the central nervous system and PNS (Table 1). The importance of vitamin C for CNS functions has been proven by the fact that the targeted deletion of the sodium–vitamin C cotransporter in mice results in widespread cerebral hemorrhage and death on postnatal day one [48,49]. It is currently known that ascorbic acid performs an antioxidative function, as it is involved in redox processes. The human body accumulates about 2–4 g of vitamin C, while, in the plasma the level of ascorbic acid, it reaches 1.4%.

The fact that vitamin C can neutralize superoxide radicals, which are generated in a large amount during neurodegenerative processes, seems to support its role in neurodegeneration. Moreover, the plasma and cellular vitamin C levels decline steadily with age, and neurodegenerative diseases are often associated with aging. An association of vitamin C release with motor activity in CNS regions, the glutamate uptake-dependent release of vitamin C, and its possible role in the modulation of N-methyl-d-aspartate receptor (NMDAR) activity, as well as ability to prevent peroxynitrite anion formation, constitute further evidence pointing toward the role of vitamin C in neurodegenerative processes [48]. Vitamin C is metabolized in the liver and kidneys, where a number of successive reactions take place, resulting in the formation of oxalic acid, which is excreted in the urine. An excess of ascorbic acid ingested with food does not have time to be metabolized and is excreted by the body in an unchanged state through the kidneys [12].

Nieves et al. (2016) in their study considered oxidative stress as one of the factors of ALS. The study involved 302 ALS patients. As a research method, the authors made an 85-item questionnaire to assess the participants’ usual diet (frequency of meals, preferred foods, specific information on admission, and intake of dietary supplements). Further, the information was statistically processed, and the average daily intake of antioxidants by the study participants was calculated. The authors analyzed the contents of vitamin C, carotenoids, and alpha-tocopherol in the diet. As a result of the study, it was found that a higher intake of vitamin C and carotenoids correlated in direct proportion with the duration and quality of life of the patients [50].

Pupillo et al. (2017) in their study studied the dietary habits of ALS patients living in three administrative regions of Italy (Lombardy, Piedmont and Valle d’Aosta, and Apulia). As the main research method, the authors used a questionnaire of the participants’ eating habits. A total of 212 people participated in the study. In the course of the study, the authors found that foods such as citrus fruits, raw vegetables, coffee and tea, and whole bread had a significant tendency to reduce the risk of ALS. This is most likely due to the lower intake of vitamins C, E, and B12 by ALS patients [51].

In another study [10], the authors studied the levels of vitamin C in the serums of healthy people and people with ALS. In addition to vitamin C, the levels of vitamins A, E, B2, and B9 and cholesterol were also taken into account. Higher levels of vitamins A and E and lower levels of vitamins B2, B9, and C were found in ALS patients compared with those in the healthy controls, and therefore, B2, B9, and C deficiency/hypovitaminosis can be considered as risk factors (predictors) of ALS [10].

Blasco et al. (2010) [49] investigated the spectrum of free radicals in the cerebrospinal fluid and the level of ascorbic acid in the serum in patients with ALS and people without neurodegenerative diseases. The authors found statistically significant high levels of ascorbic acid in ALS patients. According to the authors’ conclusions, vitamin C neutralizes free radical scavengers, modulates neuronal metabolism due to reduction by reducing glucose consumption during episodes of glutamatergic synaptic activity, and stimulates lactate uptake in neurons, which is consistent with a lower lactate/pyruvate ratio seen in ALS patients.

Spasojević et al. (2010) [52] hypothesized that the use of vitamin C might have an adverse drug reaction (ADR) in ALS patients. The authors studied the effect of vitamin C on the synthesis of hydroxide radicals in the cerebrospinal fluid of patients with sporadic ALS. The presence of ascorbyl radicals was shown in the cerebrospinal fluid of patients with ALS, although no ascorbic radicals were found in the cerebrospinal fluid of patients without ALS (control group). Moreover, the addition of hydrogen peroxide to the cerebrospinal fluid of patients with ALS provoked the further formation of ascorbyl and hydroxyl radicals. According to the authors, vitamin C can have not only protective antioxidant effects in ALS, but it can also paradoxically cause a prooxidant effect, which is probably important to take into account when using high doses of vitamin C.

Nagano et al. (2003) investigated the effect of vitamin C in the diet in an animal model of ALS (mice). Vitamin C was prescribed before and after the onset of the disease. Mice that received vitamin C before the onset of the disease had a higher survival rate compared to the control group (>62%). However, other research results [53] did not prove the effect of vitamin C on the average age of ALS onset. The introduction of vitamin C after onset of the disease did not significantly increase the survival rate of patients. In addition, it was found that the infusion of vitamin C as a monotherapy or in combination with glutathione ethyl ester did not prevent the degradation of motor neurons in rats [53,54].

Fitzgerald et al. (2013) analyzed five large prospective studies (involving about 1100 patients with ALS) and showed that even long-term supplementation with vitamin C, or a high intake of vitamin C with food, does not affect the risk of developing ALS in humans [9].

**Figure 8:**
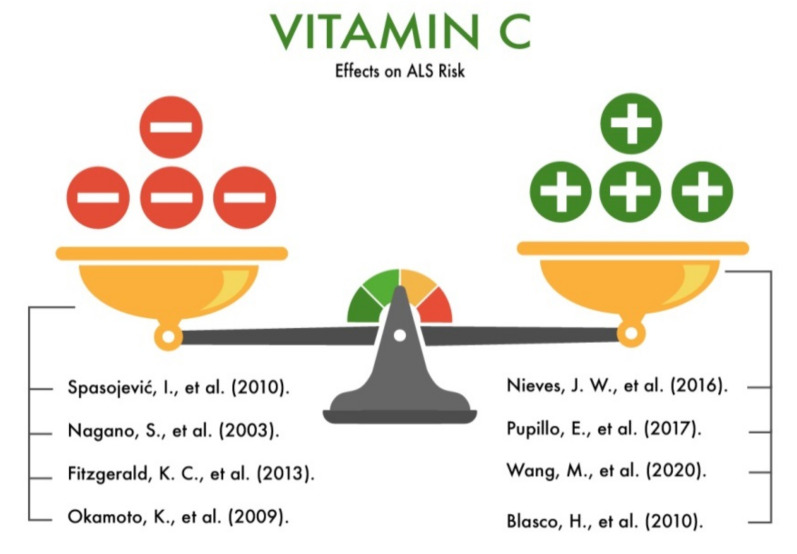
The effect of vitamin C on the risk of developing amyotrophic lateral sclerosis (ALS).

Okamoto et al. (2009) investigated the relationship between the consumption of vegetables, fruits, and antioxidants and the risk of developing ALS in Japanese people aged 18 to 21 years. The authors showed that a higher consumption of fruits and/or vegetables led to a significant reduction in the risk of ALS [55]. However, the authors did not find a statistically significant relationship between the vitamin C dose and ALS risk. Thus, despite the large number of studies devoted to the study of the effect of vitamin C on motor neurons and the risk of developing ALS, its role requires further study in various ethnic groups of patients (Figure 8).

### 3.9. Vitamin E

Vitamin E (alpha-tocopherol, tocopherol, and tocotrienol) is a fat-soluble essential vitamin that mainly enters the human body from food (Table 2) [67]. In the gastrointestinal tract, tocopherol is hydrolyzed by lipase and esterase. Then, it is absorbed in the upper parts of the small intestine and enters the bloodstream through the lymphatic system. Vitamin E is essential for the normal functioning of the central nervous system. It is a major fat-soluble antioxidant that protects the integrity of neuronal cell membranes by inhibiting lipid peroxidation. Orally taken vitamin E reaches the cerebrospinal fluid and brain. It has been shown that eight substances have an activity of vitamin E: alpha-, beta-, gamma-, and delta-tocopherol and alpha-, beta-, gamma-, and delta-tocotrienol. However, more often, the synonym for alpha-tocopherol is used to denote the term vitamin E [67].

Sen et al. (2004) showed that alpha-tocotrienol is many times more effective than alpha-tocopherol in protecting neurons from the toxicity caused by glutamate and other neurotoxins. In addition, the authors have shown that dietary supplements containing alpha-tocotrienol do indeed reach the brain and have powerful, neuroprotective effects. However, studies of ALS in ALS patients were conducted using alpha-tocopherol but not alpha-tocotrienol [68]. It is possible that, in the studies we analyzed, the term vitamin E was used for both alpha-tocopherol and alpha-tocotrienol. Tocopherol is known for its antioxidant properties, but its biochemical and physiological functions in regulating the activity of the central nervous system are being actively studied and expanded (Table 1) [68]. That is why many studies have been devoted to the study of the role of this vitamin in the development of ALS.

Fang et al. (2015) [69] in their meta-analysis considered the risk factors for ALS; among which, the nutritional factors were also studied. The authors concluded that one of the best-studied relationships between dietary factors and ALS is the inverse relationship between a higher intake of antioxidants and a lower risk of ALS. The authors cited a study in which regular vitamin E supplementation was associated with a lower risk of ALS [70], and longer vitamin E intake was associated with a lower risk of ALS in a large study combining separate data from five cohorts [71].

Veldink et al. (2006) examined the relationship between the dietary intake of antioxidants and the risk of ALS, adjusted for confounding factors. The study involved 132 patients. The patients were asked to fill in three identical questionnaires. One questionnaire was completed by the ALS patients. The other two questionnaires were to be completed by the participants in the control groups (not a spouse and not a partner, with the age difference not more than 5 years and, presumably, of the same gender). The questionnaire included two sections. The first section included questions about age, gender, educational level, smoking, and anthropometric characteristics; the second section consisted of questions about dietary habits. As a result, the premorbid total energy consumption was similar in the two groups, which corresponded to similar premorbid body mass index (BMI) levels. The intake of vitamin E and polyunsaturated fatty acids (PUFA) was noticeably lower in ALS patients than in the control group. The study found that an average vitamin E intake of <18–25 mg/day was associated with a 60% lower risk of ALS. The highest intake of vitamin E (>22 mg/day) was associated with a 50% lower risk of ALS compared to the lowest intake (<18 mg/day). Vitamin E intakes in the range of 18–22 mg were associated with a 60% lower risk of ALS. The highest intake of vitamin E (22 mg per day) was associated with a 50% lower risk of ALS compared to the lowest intake (18 mg) [72].

Richard (2020), in his review, considered oxidative stress as one of the causes of ALS and the neuroprotective function of vitamin E. The author analyzed several studies conducted in an animal model in which vitamin E supplementation promoted the survival of motor neurons when exposed to various neurotoxins and, in addition, the authors of clinical cases of ALS and the effect of vitamin E on the prognosis of the disease. The author paid special attention to one clinical case in 1940: after two months of taking vitamin E, the condition of one patient with ALS improved significantly. Specifically, before taking vitamin E, the patient complained of hyperreflexia, tongue atrophy, and an inability to walk. After a course of vitamin E, the patient began to walk with assistance, and the muscular strength of the extremities partially recovered [73,74,75].

Okamoto et al. (2009) conducted a study involving 153 ALS patients. All patients underwent a questionnaire that included questions about lifestyle, social factors, and dietary habits before and during the illness. Nutrient intake estimates were calculated using Japan’s Standard Food Composition Tables [96]. A higher intake of vitamin E was reported to be associated with a lower risk of ALS. However, there was no statistically significant association [55].

Ascherio et al. (2004) prospectively studied whether people who regularly use antioxidant vitamin E supplements have a lower risk of developing ALS. The study was conducted among participants in Cancer Prevention Study II (CPS-II). All participants completed a survey that included questions about the current intake of vitamin A, vitamin C, vitamin E, and multivitamins. Participants were asked to report how many times they had used each supplement in the last month and the number of years of use. The mortality rate from ALS among long-term vitamin E users was 62% lower than among those who did not. According to the results of the study, participants who did not take vitamins C and E and multivitamins and participants who regularly took vitamin C but did not take vitamin E were equally not at a reduced risk of ALS [70].

**Figure 9:**
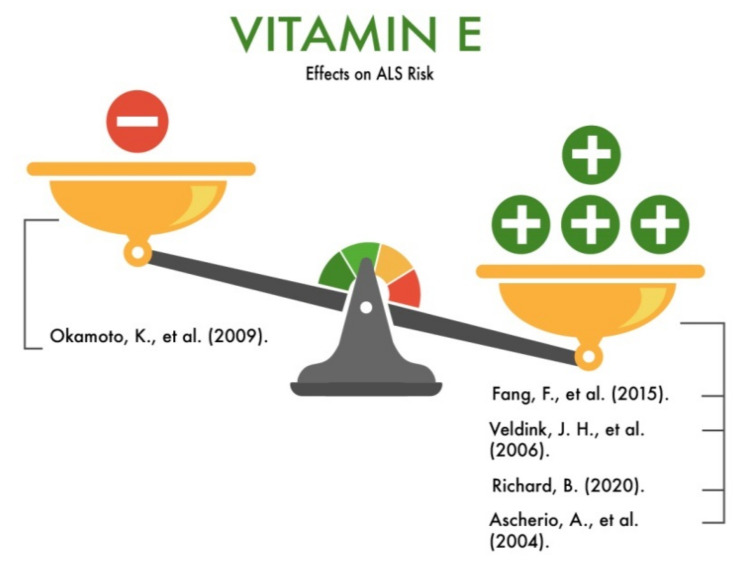
The effects of vitamin E on the risk of developing amyotrophic lateral sclerosis (ALS).

Therefore, vitamin E can be considered as a protector for the development and progression of ALS (Figure 9).

### 3.10. Vitamin D

Vitamin D (ergocalciferol and cholicalciferol) is a secosterol that is produced in the skin under the influence of the ultraviolet spectrum of sunlight or enters the body from food, biologically active additives (dietary supplements), or monocomponent or multicomponent vitamin–mineral complexes [56]. Vitamin D is involved in many biological processes in the CNS, PNS, and skeletal muscle (Table 1) [57,58]. It has been shown that vitamin D plays an important role in the development and protection of the central nervous system, and vitamin D also plays a role in the development of neurodegenerative diseases. Thus, vitamin D can modify the risk, prognosis, and response to therapy in patients with various neurodegenerative diseases, including ALS. In addition, vitamin D promotes cerebral activity in both embryonic and adult brains, helping to connect the neural circuits that are responsible for locomotion, emotion, and behavior [59]. For this reason, in recent years, the non-skeletal functions of vitamin D have been actively studied, including its protective and predictive role in the development of neurodegenerative diseases, including neuroprotection.

To date, there are many studies examining the level of vitamin D in the blood serum and its relationship with the development of motor neuron diseases. Specifically, the studies by Camu et al. (2014) and Karam et al. (2013) confirmed the protective role of vitamin D [60,61]. However, we found three studies that reported questionable results [62,63,64], and one study that did not confirm the role of vitamin D in ALS [65]. The study of Camu et al. (2014) described a cellular model of ALS (isolated motoneurons of the spinal cord of transgenic mice embryos) and ALS patients. The authors discovered, for the first time, that all neurons expressing GFP under the control of the motor neuron-selective promoter Hb9 (Hb9: GFP) express a vitamin D receptor (VDR), which, in turn, binds to vitamin D to perform its biological function. Thus, the vitamin D levels are important for the normal functioning of the spinal cord motor neurons. Next, the authors investigated whether the active metabolite 1.25 (OH) 2D3 could induce neurite outgrowth in embryonic motor neurons. However, there was no statistically significant effect of vitamins D2 or D3 or the active metabolite 1.25 (OH) 2D3 on the elongation of motoneurites [60,66].

Karam et al. (2013) conducted a study involving 37 patients with ALS. The serum level of the active metabolite of vitamin D was determined in all the subjects. In addition, the following factors were taken into account: age, gender, time of ALS symptoms onset, time of diagnosis, the presence or absence of depression, smoking, multiple head trauma, accompanying illnesses, and taking nutritional supplements with vitamin D and other vitamins. Additionally, the authors took into account the pathogenetic therapy of ALS by taking riluzole. The Amyotrophic Lateral Sclerosis Functional Rating Scale (ALSFRS-R) was used to monitor the progression of ALS. In 81% of patients, the serum levels of 1.25 (OH) 2D3 were below 30 ng/ml (vitamin D deficiency), and 43% had levels below 20 ng/ml (vitamin D deficiency) [61].

Lanznaster et al. (2020) published a meta-analysis of studies performed in patients with ALS, which included clinical trials, cohort studies, and case–control studies. The authors used various tools to analyze the risk of bias (RoB 2, ROBINS-I, QUIPS, DL, and JG). As a result of the information processing, the following data were analyzed: numerical values of the serum vitamin D levels for patients with ALS, an informed statistical index, a statistical index of the relationship between the level of vitamin D and functional score and its confidence interval (CI), a statistical indicator of the correlation between the vitamin D levels in patients receiving dietary supplements (dietary supplements), and the functional assessment. The authors reviewed three types of comparisons: vitamin D levels in ALS patients versus non-ALS/healthy controls, the relationship between the vitamin D levels and survival in ALS patients, and the survival of patients receiving dietary supplements containing vitamin D and not receiving such dietary supplements. As a result, the authors obtained rather contradictory results in the studied articles, which do not allow us to speak unambiguously about the prognostic role of the serum vitamin D levels in the survival of ALS patients [62].

Yeshokumar et al. (2015) analyzed studies on the neuroprotective role of vitamin D in cultured motor neurons in vitro and showed that vitamin D enhanced the action of the glial brain neurotrophic growth factors and protected the motor neurons from Fas-induced cell death in the culture [63].

Blasco et al. (2015) conducted a systematic review of the clinical trials, cohort, and case–control, which reported the level of 1.25 (OH) 2D3 as a putative biomarker for the diagnosis or prognosis of ALS, or the effect of nutritional supplements with vitamin D contents in patients with ALS. As a result, ambiguous results were obtained. The authors found that the serum vitamin D levels in the ALS patients were slightly lower than in the control group, but additional environmental factors were not taken into account. However, the relationship between the level of this vitamin D metabolite and the rate of progression of ALS has not been confirmed. Additionally, contradictory results have been obtained regarding the survival and effects of supplementation with vitamin D [64].

**Figure 10:**
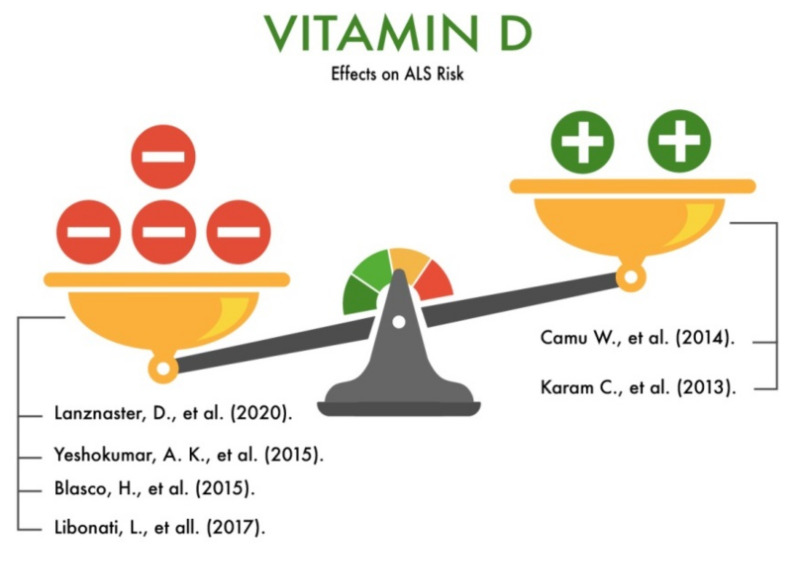
The effect of vitamin D on the risk of developing amyotrophic lateral sclerosis (ALS).

Libonati et al. (2017) conducted a retrospective study involving ALS patients and healthy people. The authors compared the serum levels of 1.25(OH)2D3 in ALS patients and in healthy volunteers. However, no statistically significant differences were found [65]. Thus, despite the large number of studies devoted to studying the effects of vitamin D on motor neurons and the risk of developing ALS, the translation of their results into real clinical practice is difficult, since a large number of the studies we analyzed did not confirm the protective role of vitamin D on the development and progression of ALS (Figure 10).

### 3.11. Cholesterol

Cholesterol is one of the most important sterols that is synthesized in the human body, mainly in the liver. Cholesterol is an important component of cell membranes, a precursor for the synthesis of steroid hormones, vitamin D, and bile acids. Cholesterol is transported in the plasma mainly in the form of low-density lipoproteins (LDL). The main route of its removal from tissues to the liver is high-density lipoproteins (HDL) with a subsequent excretion in bile [76]. Due to the important function of cholesterol, it is critical to maintain adequate levels of it. However, an elevated cholesterol level is a predictor of many diseases, including neurodegenerative ones. Some studies of high-fat diets that we analyzed showed a reduction in the risk of developing ALS by at least 34%, 56% [77], and even 50–60% [72] in people with ALS. However, a number of studies have shown that a diet high in cholesterol and LDL cholesterol contributed to an acceleration in the rate of disease progression and a decrease in the survival rate of patients with ALS [78].

It is assumed that a diet high in HDL reduces the risk of developing ALS, while a diet high in LDL cholesterol increases this risk [51]. In particular, Huisman et al. (2015) [78] studied fatty acids and cholesterol using a differentiated high-fat diet. The authors found a correlation between the intakes of both nutrients (*p* = 0.95 for trans fatty acids; *p* = 0.73 for cholesterol) with a reduced risk of adverse ALS. In contrast to these fatty nutrients, the intake of the plant proteins, polysaccharides, dietary fiber, and flavonoids, which were analyzed together in a multivariate model of these nutrients, were shown to be statistically significantly associated with the risk of ALS. A high premorbid BMI was associated with a reduced risk of ALS (*p* = 0.01).

**Figure 11:**
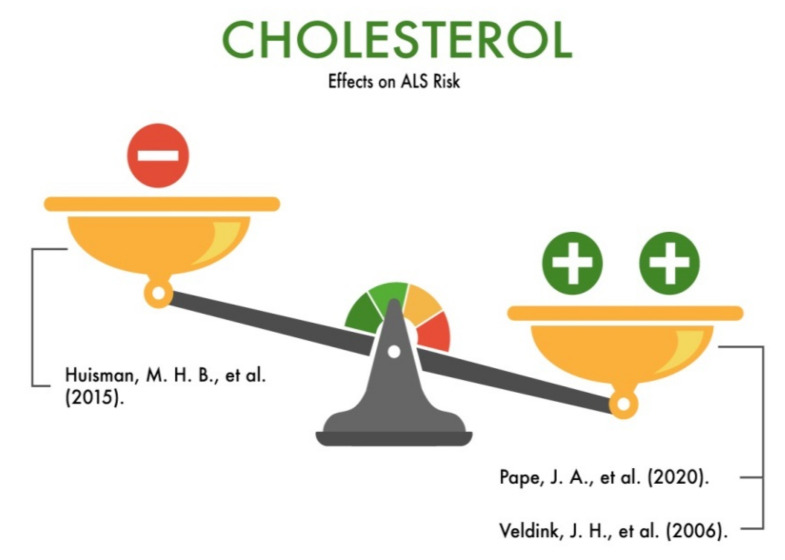
The effect of cholesterol on the risk of developing amyotrophic lateral sclerosis (ALS).

Some authors believe that a high fat intake is associated with a high risk of ALS [79]. Other authors showed alternative results: a decrease in the risk of ALS in people with a high fat intake [80]. Finally, a number of authors have demonstrated that a fat intake does not statistically significantly affect the course of pre-symptomatic ALS [81]. Thus, many authors support the hypothesis that a high fat intake in patients with pre-symptomatic ALS is not compensatory for increasing the energy metabolism, although it can partially increase it. There are contradictory opinions about the need for new research on the role of high-calorie and ketogenic diets in ALS in the future (Figure 11).

### 3.12. Polyunsaturated Fatty Acids

Polyunsaturated fatty acids (PUFAs) include two sets of fatty acids: omega-6 and omega-3. PUFAs have hydrophilic and hydrophobic properties due to their structure (hydrophilic head and hydrophobic tail) [82]. Due to these properties, PUFAs perform a number of important biological actions; among which, the most interesting are the inhibition of inflammatory processes and a decrease in the secretion of proinflammatory cytokines [83]. Additionally, PUFAs are responsible for a number of important functions in the CNS and PNS (Table 1) [84].

The content of PUFAs in the brain is high. Once PUFAs are released from the cell membranes of neurons, they can participate in signaling, either directly or after enzymatic conversion into various bioactive derivatives (mediators). PUFAs and their mediators can regulate various processes in the brain, including neurotransmission, cell survival, and neuroinflammation [85]. Therefore, the role of PUFAs in the development of ALS has been actively studied in recent years [86].

Veldink et al. (2006) conducted a study involving 132 ALS patients and a control group of 220 healthy people. The study was conducted in 2001 to 2002. The inclusion criteria for patients with ALS in the study were: a clear, provisional, or possible diagnosis of ALS in accordance with the El Escorial criteria and the absence of a positive family history (absence of ALS in relatives). The authors used a questionnaire to assess the diet and intake of nutrients under investigation, including PUFAs. Next, a multivariate logistic regression analysis was performed, adjusted for confounding factors (gender, age, educational level, energy consumption, BMI, and smoking). The multivariate analysis showed that the highest daily intake of PUFAs (0.32 g per day) was associated with a 60% lower risk of ALS compared with the lowest daily intake (0.25 g per day) [72].

Kim et al. (2005) showed that long-chain PUFAs promoted the formation of aggregates of mutated SOD1 in an in vitro experiment. This allowed the authors to hypothesize that PUFAs reduce the risk of ALS [87].

Fitzgerald K. C. et al. (2014) conducted an extensive analysis involving 995 ALS patients. The participants were given questionnaires that included questions about the frequency of meals and the habitual consumption of each food product. Then, the amount of nutrients was estimated by multiplying the frequency of consumption by the indicated serving size. These surveys provided information on the energy and macronutrient intake, including the intake of saturated, monounsaturated, and certain dietary PUFAs. Information on other covariates of interest was also taken into account, including smoking status, height, weight, educational level, and physical activity. As a result, the total consumption of ω-3 PUFAs was associated with a 34% reduction in the ALS risk in the multivariate model compared to the highest and lowest quintiles (multivariate pooled relative risk (RR) 0.66; 95% CI, 0.53–0.81; *p* < 0.001 for the trend). However, the total intake of ω-6 PUFAs was not associated with the ALS risk (multivariate pooled RR when comparing highest to the lowest quintile, 0.88; 95% CI, 0.72–1.08; *p* = 0.22 for the trend) [88].

González De Aguilar [89] conducted a meta-analysis that examined the main biomarkers of ALS, including the serum PUFA levels. Thus, in one study reviewed by the author, the neuroprotective role of PUFAs was proven [88]. In contrast, other studies in this meta-analysis did not demonstrate an association between a high premorbid PUFAs intake with an increased risk of ALS [78]. However, another cited study found that higher levels of palmitoleic/palmitic fatty acids correlate with a better functional status and are associated with longer survival in ALS patients [90].

O’Reilly et al. (2019) conducted a large study involving both ALS patients and healthy subjects. The authors studied the level of PUFAs in the human blood. The following factors were also analyzed: growth, weight, smoking experience, the level of education, physical activity, and the presence or absence of diabetes. The authors did not find a statistically significant association between the serum levels of the general PUFAs, n-3 and n-6 PUFAs, eicosapentaenoic acid, or docosapentaenoic acid and ALS. However, an elevated level of plasma α-linolenic acid (ALA) in men was associated with a lower risk of ALS in the age-adjusted analysis (OR = 0.21). The study also found that, in women, increased plasma arachidonic acid levels were associated with a higher risk of ALS (OR = 1.65). Multivariate adjustments, including correlated PUFAs, did not change the results for ALA and arachidonic acid. Additionally, in men and women, a higher level of docosahexaenoic acid (DHA) in the plasma was found, which was associated with a high risk of ALS (OR = 1.56). However, in multidimensional models, this association was confirmed only in men [91].

**Figure 12:**
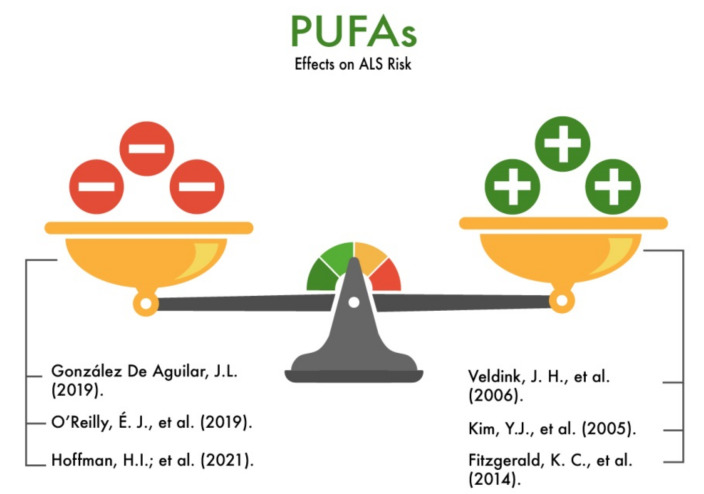
The effect of polyunsaturated fatty acids on the risk of developing amyotrophic lateral sclerosis (ALS).

Hoffman et al. (2021) conducted a study involving patients with ALS and healthy subjects. All participants completed a questionnaire that analyzed the frequency of consumption of seafood containing PUFAs. In addition, the authors analyzed the concentration of mercury in consumed seafood. Additionally, other factors were taken into account: gender, family history of ALS, and smoking status. According to these study results, the consumption of mercury and omega-3 PUFAs with seafood is not associated with an increased risk of ALS [92]. Therefore, despite a large number of studies, the protective effect of PUFAs on the risk of developing or reducing the rate of degeneration of motor neurons in ALS requires further study (Figure 12).

### 3.13. Urates and Purines

Uric acid and its salts (urates) and purines are considered powerful antioxidants that can affect the redox processes in the CNS and PNS (Table 1). For this reason, studies are currently being undertaken to investigate the urate and purine effects on the course and prognosis of many neurological diseases, including ALS [93].

The studies covering the problem of the effects of urates and purines on the risk of developing ALS showed conflicting results. In particular, O’Reilly (2017) showed that a diet high in urates and purines moderately increases the risk of developing ALS [94]. At the same time, Paganoni et al. (2017) demonstrated that the consumption of foods with a high content of urates and purines in patients with ALS has a positive effect on the course of the disease, increasing the median survival rates [95]. Pupillo et al. (2017) found a statistically significant trend in the increased risk of ALS in individuals with a high consumption of red meat (odds ratio (OR) = 2.96).

**Figure 13:**
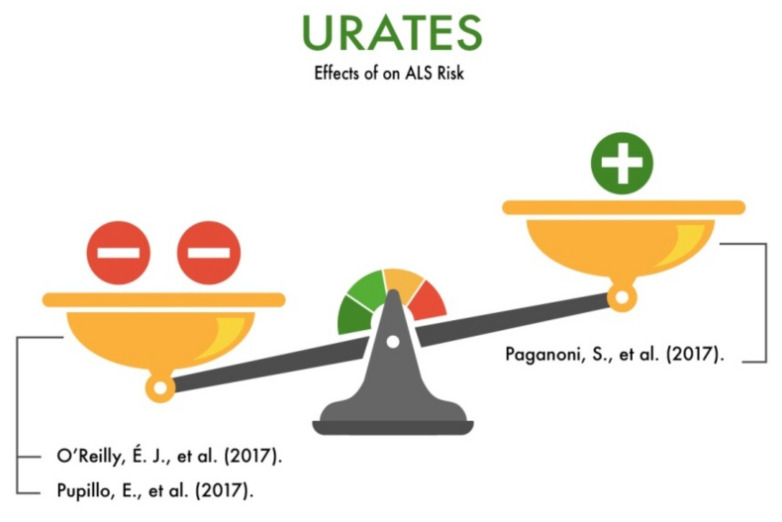
The effect of urates and purines on the risk of developing amyotrophic lateral sclerosis (ALS).

However, it is not known whether these studies can be translated into other ethnic groups of ALS patients [51]. Thus, despite the promising results of the studies we analyzed, the roles of urates and purines in the development of ALS and degeneration of motor neurons need further study (Figure 13).

## 4. Discussion

**Figure 14:**
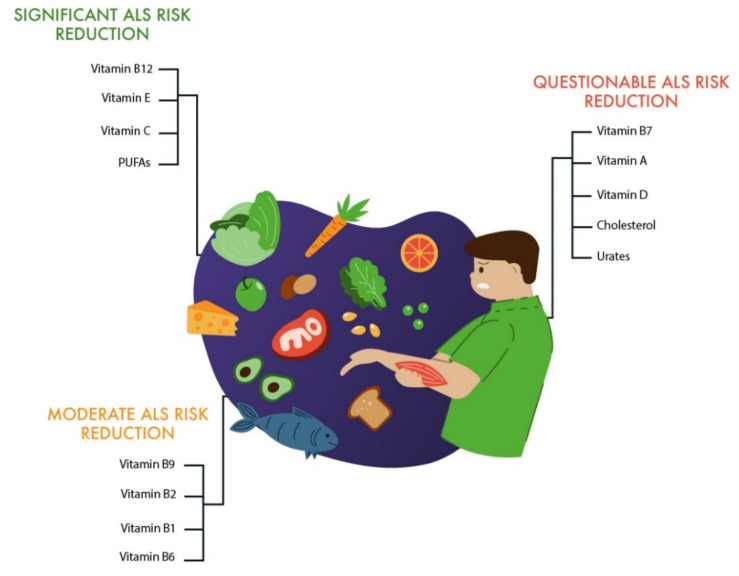
The role of nutrients in reducing the risk of developing and the progression of amyotrophic lateral sclerosis (ALS).

We analyzed 39 studies, including seven meta-analyses. Of all the studies analyzed, seven studies were performed on an animal model of ALS, and 34 studies were performed on patients with ALS. The works were mainly devoted to the study of the following nutrients (Table 1): vitamin A (carotenoids)—two studies (including one study with a significant association and one study with an ambiguous association); vitamin B1 (aneurin and thiamine)—two studies (with a significant association in all the studies); vitamin B2 (riboflavin)—one study with a significant association; vitamin B6 (adermine and pyridoxine)—one study with a significant association; vitamin B7 (B8, H, and biotin)—two studies (including one study with a significant association and one study with an ambiguous association); vitamin B9 (folic acid)—two studies (with a significant association in all the studies); vitamin B12 (cyanocobalamin)—five studies (including four studies with a significant association and one study with no association found); vitamin C (ascorbic acid)—eight studies (including four studies with a significant association, two studies with an ambiguous association, and two studies with no association found); vitamin D (ergocalciferol and cholecalciferol)—six studies (including two studies with a significant association, three studies with an ambiguous association, and one study with no association found); vitamin E (alphatocopherol)—five studies (four with a significant association and one with a dubious association); food with a high cholesterol content—three studies (including two studies with a significant association and one study with no association found); food with a high content of polyunsaturated fatty acids (PUFAs)—six studies (including three studies with a significant association, one study with an ambiguous association, and two studies with no association found); and food and drinks high in uric acid and purines—three studies (including one study with a significant association and two studies with no association found) (Figure 14).

According to the publications we studied, the most significant protectors of ALS development were the following vitamins: vitamin B12 and vitamin E > vitamin C > vitamin B1, vitamin B9 > vitamin D > vitamin B2 and vitamin B6 > vitamin A and vitamin B7 (Table 1 and Table 2).

Our data partially correlated with the results of another study [10], which showed the following protective role of vitamins that reduce the risk of ALS: vitamin A > vitamin B 12 > vitamin E > vitamin B1 > vitamin D > vitamin C > vitamin B9 > vitamin B2.

In addition, this review indicates that foods with a high content of cholesterol and PUFAs, foods and beverages with a high content of uric acid salts (urates), and purines play significant roles in the development of ALS (Table 2 and Table 3).

In recent years, various ALS patient-oriented diets promising at both the pre-symptomatic stage and at the advanced stages of the disease have been actively studied. The main goal of developing such diets is to slow down the pathological process of degeneration of motor neurons in patients with ALS and increase the age of onset and/or prevention of ALS in the ALS patients’ relatives (in the case of familial cases of the disease). Specifically, a gluten-free diet [97,98] and a ketogenic diet [99] are considered as promising diets. Studies on gender differences in susceptibility to different diets for ALS in men and women are of great interest, since a number of studies have shown that men have a higher risk of developing ALS and a more severe course of the disease [100,101].

Our thematic review of the studies on the role of nutrients in ALS sought to assess the roles of vitamins, cholesterol, urates, and purines. Vitamin B12 is the most promising for inclusion in the ALS treatment protocol. The roles of other B vitamins are important but need to be clarified. Undoubtedly, the roles of antioxidants (vitamins E and C) are great. As a result of the analysis, it was shown that a vitamin E intake is associated with a decrease in the risk of ALS in most studies [70,71], but not all of these studies showed a slower progression of the disease or an improvement in the quality of life of the patients. In general, most of the vitamins we analyzed have an additive effect on the development and progression of ALS (Figure 14).

Nutritional supplements (dietary supplements) should be included in the treatment plan for patients with ALS both at the preclinical and clinical stages of the development of the disease as a disease-modifying therapy. However, the choice of nutritional supplements and diet should be made on a personalized basis, taking into account the results of the laboratory diagnostics. Therefore, to select vitamins or multivitamin complexes, it is necessary to study the levels of their active metabolites in the blood serum of patients with ALS before the administration of disease-modifying therapy, as well as their dynamics. This is important, because a lack of vitamins and nutrients, along with their excess, can have a negative effect on the body of a patient with ALS.

We believe that self-medication or the uncontrolled intake of vitamins and nutrients for ALS is unacceptable. From the standpoint of personalized medicine, it is also important to take into account the possibility that patients have background (comorbid) genetically determined metabolic disorders of the above nutrients. This may partly explain the conflicting results of the previous epidemiological and cross-sectional clinical studies. In general, the approach to the adjustment of diets for ALS needs to be careful and personalized.

## 5. Limitations

There are several limitations in our thematic research. We studied only English language and Russian language publications. It is likely that taking different nutrients can have a variable effect on the decrease in the risk of development ALS. Men and women may respond differently to the nutrients in ALS. Further studies are needed to study the gender effects of nutrients in monotherapies and polytherapies in patients with ALS.

The role of nutrigenetics in the absorption, transport, accumulation, metabolism, and excretion of nutrients is very important. In particular, single-nucleotide variants (SNVs)/polymorphisms of candidate genes encoding ascorbate transporter proteins may affect the efficacy and safety of vitamin C, which is the most-studied nutrient in ALS. The contradictory results of previously conducted clinical studies can be explained not only by different methodological approaches but also by nutrigenetics in different ethnic groups of patients. Further studies are needed to investigate the effects of carriage of SNVs/polymorphisms of genes encoding serum and urinary metabolites of the main nutrients on the serum and urine levels in patients with ALS. The role of nutrigenetics and a personalized approach to prescribing the most promising nutrients to patients with ALS are very important for understanding their effectiveness and safety.

Finally, the role of microbiota in the synthesis of nutrients and the development of neurodegenerative diseases has been considered in recent years. This seems important for new, thematic reviews in the future and the translation of the results of basic research into real clinical practice.

## 6. Conclusions

The present review of the role of nutrients as predictors of ALS emphasizes the importance of developing a specialized diet for patients at various stages of ALS development (preclinical stage, disease onset, and advanced stage), as well as for people with familial ALS. At the same time, the approach to the choice of nutrients for patients with ALS should be careful and personalized; it should be negotiated with the consulting physician. Self-medication with multivitamins and dietary supplements is unacceptable [8].

It should be recognized that, at present, there is no universal diet or a universal algorithm for vitamin therapy for ALS. It is necessary to plan large, multicenter ALS studies (both in humans and with the use of an animal model) with a unified study design and emphasis on both candidate genes responsible for the development of ALS [102,103] and candidate genes responsible for comorbid genetically determined disorders of the nutrient metabolism in ALS patients.

## Figures and Tables

**Table 1:** The role of vitamins and other nutrients in the functioning of the nervous system and the development of ALS.

Nutrient	Function in the CNS	Role in the Development of ALS	Authors
Vitamin A (retinol)	Regulation of oxidation-reduction processes; regulation of protein synthesis; participation in redox processes (neutralization of free oxygen radicals); participation in the development of cells, providing sensitivity to hormones and growth stimuli; regulation of normal growth and differentiation of cells of the embryo and young organism; regulation of division and differentiation of rapidly dividing tissues, including cells of the immune system.	Is likely to reduce the risk of ALS	[7,8,9,10]
Vitamin B1 (thiamine)	Participation in oxidative metabolism; neuroprotection (reduction of neuroinflammation and neurodegeneration); participation in carbohydrate metabolism and associated energy, fat, protein, and water-salt metabolism; regulation of the activity of the central nervous system; optimization of the impact on cognitive activity; participation in the neutralization of xenobiotics (protection from the toxic effects of alcohol and nicotine); slowing down the aging process; imitation of the action of acetylcholine on neurons; participation in the exchange of zinc and manganese.	Is likely to reduce the risk of ALS	[11,12,13,14,15]
Vitamin В2 (riboflavin)	Participation in the oxidation of fatty acids, succinic acid, and amino acids; participation in the regulation of tissue respiration and cell growth processes; participation in redox reactions as an antioxidant; participation in energy exchange; participation in the processes of assimilation of iron; protection of the retina from the harmful effects of ultraviolet radiation; neuroprotection; participation in tissue regeneration; participation in the formation of red blood cells and antibodies; influence on pain and tactile sensitivity; excitability of the CNS and PNS.	Is likely to reduce the risk of ALS	[10,12,16,17,18]
Vitamin В6 (pyridoxine)	Participation in the synthesis of serotonin; participation in the circadian cycle regulation; vitamin B12 cofactor; coenzyme of a large group of pyridoxal enzymes (transfer of amino groups, decarboxylation of amino acids, and hydroxylation); decrease in the excitability of the CNS.	Is likely to reduce the risk of ALS	[12,13,19,20,21,22,23,24,25]
Vitamin В7 (biotin)	Energy function and regulation of energy metabolism (adenosine triphosphate (ATP) production); participation in carboxylation reactions; participation in the synthesis of purines; participation in the metabolism of fatty acids; participation in the neutralization of xenobiotics (detoxification); participation in the circadian cycle regulation; influence on cognitive functions and attention; participation in regeneration processes; reduction of neuroinflammation processes; coenzyme participating in the reaction of the CO_2_ transfer to organic compounds; interaction with insulin (stabilization of blood glucose levels); participation in the production of glucokinase; slowing down the aging process; participation in the neutralization of xenobiotics (detoxification).	Is likely to reduce the risk of ALS	[12,26,27,28,29,30,31,32]
Vitamin В9 (folic acid)	Cyanocobalamin cofactor; participation in the formation of erythrocytes and leukocytes; participation in the processes of iron metabolism; participation in the synthesis of nucleotides and DNA; participation in the resynthesis of methionine from homocysteine (together with vitamin B12); participation in the synthesis of choline, creatine, and adrenaline; influence on lipid metabolism and blood cholesterol level; regulation of cell division and participation in fetal development; participation in neuroimmune reactions and neuroprotection; participation in the synthesis of purines and pyrimidines necessary for the formation of the genetic code (DNA, RNA-replication processes); participation in the exchange of glycine and serine, methionine, and histidine; participation in the biosynthesis of dopamine, norepinephrine, and serotonin; slowing down the aging process and protection against oncopathology.	Is likely to reduce the risk of ALS	[10,12,23,24,33,34,35,36,37,38,39,40]
Vitamin В12 (cyanocobalamin)	Participation in the regulation of the folate cycle (regulation of homocysteine levels); cofactor of vitamin B9 (folic acid); participation in the regulation of amino acids and fatty acids (pro-pionic acid); participation in the regulation of growth and differentiation of neurons (active influence on cell division); participation in the regulation of the formation of the myelin sheath; influence on cognitive and emotional-volitional functions; participation in the regulation of the balance function; participation in the conversion of folic acid derivatives necessary for the synthesis of DNA and RNA nucleotides; participation in the regeneration of methionine; participation in the metabolism of polyunsaturated fatty acids with an odd number of carbon atoms; influence on the exchange of amino acids with a branched side chain (methionine, isoleucine, trionine, and valine); participation in the synthesis of adrenaline, acetylcholine; influence on the level of cholesterol in the blood; regulation of CNS excitability; participation in the formation of erythrocytes; slowing down the aging process and protection against oncopathology.	Reduces the risk of ALS	[12,23,36,37,41,42,43,44,45,46,47]
Vitamin C (ascorbic acid)	Participation in redox processes (protection from oxygen free radicals); participation in the synthesis of proteins (amidation of peptides); participation in the synthesis of myelin; synaptic potentiation; neuroprotection (protection from the action of excitatory neurotransmitters such as glutamate); participation in regeneration processes; participation in energy processes; participation in the absorption of calcium and iron; participation in the regulation of the neuroimmune response (influence on resistance to viruses, bacteria, and parasites); slowing down the aging process and protection against oncopathology; enhancing the effect of adrenaline (anti-stress effect); participation in the regulation of emotional reactions, cognitive functions; participation in the exchange of cholesterol; participation in the synthesis of collagen; impact on mental and physical performance; influence on the function of equilibrium; increasing resistance to unfavorable environmental factors (infections, exposure to low doses of chemicals, ionizing radiation, and reduction of undesirable reactions of a number of drugs).	Reduces the risk of ALS	[9,10,12,48,49,50,51,52,53,54,55]
Vitamin D (ergocalciferol, cholecalciferol)	Regulation of blood calcium phosphate levels; participation in the regulation of the neuroimmune response; influence on the proliferation and differentiation of neurons; influence on synaptic transmission of nerve impulses mediated by calcium current; neurotrophic function; neuroprotective function; influence on neurotransmission and synaptic plasticity; influence on synaptogenesis; participation in the regulation of aging processes, including the death of neurons (apoptosis).	Is likely to reduce the risk of ALS	[56,57,58,59,60,61,62,63,64,65,66]
Vitamin E (alphatocopherol)	Decrease in neuroinflammation and oxidative stress; participation in redox processes (antioxidant function, preventing lipid peroxidation, and reducing free radical reactions in rapidly dividing cells); protection of vitamin A from oxidation, which contributes to the growth of the stimulating activity of vitamin A; participation in the regulation of tissue regeneration; participation in the regulation of neuronal excitability and neuroinflammation; participation in the regulation of aging processes; participation in the synthesis of hormones; slowing down the aging process and protection against oncopathology; participation in maintaining the normal functioning of skeletal muscles; reduction of muscle tissue degeneration processes; participation in energy metabolism and thrombogenesis; participation in the formation of collagen and elastin fibers (strengthening the walls of cerebral vessels); participation in the formation of hemoglobin; reduction of muscle tissue degeneration processes.	Reduces the risk of ALS	[55,67,68,69,70,71,72,73,74,75]
LDL Cholesterol	Proatherogenic activity; transport of cholesterol from the liver to the nervous tissue; transfer of unsaturated fatty acids (diene, triene) and polyunsaturated fatty acids as part of cholesterol and triglyceride esters.	Is likely to increase the risk of ALS	[51,72,76,77,78,79,80,81]
HDL Cholesterol	Antiatherogenic effect; removal of excess cholesterol from nerve tissue cells and from the surface of other lipoproteins; the supply of proteins and esterified cholesterol to lipoproteins (increasing their stability); antioxidant effect on low density lipoproteins; capture of cholesterol from macrophages (prevention of atherosclerotic vascular lesions); reverse transport of cholesterol from the nervous tissue to the liver (excretion of cholesterol from the body as part of bile acids); prevention of the capture of particles saturated with cholesterol by cells (high affinity with *apolipoprotein* E- receptors and *apolipoprotein* B-receptors).	Is likely to reduce the risk of ALS	[51,72,76,77,78,79,80,81]
Polyunsaturated fatty acids (PUFAs)	Plastic function (substrate for the formation of phospholipids, glycoproteins, the formation of a cell membrane, a sheath of nerve fibers); elimination of cholesterol from the body; protective action; participation in the exchange of vitamins B1 and B6; increased elasticity and decreased vascular permeability (antiatherogenic function); biosynthesis of prostaglandins; acceleration of the transmission of nerve impulses.	Are likely to reduce the risk of ALS	[72,78,82,83,84,85,86,87,88,89,90,91,92]
Urates and purines	Participation in the synthesis of nucleic acids (participation of pyrimidine and purine in the composition of pyrimidine and purine bases).	Are unlikely to have a role in increasing/reducing the risk of ALS	[51,93,94,95]

**Table 2:** Foods containing nutrients reducing the risk of developing amyotrophic lateral sclerosis (ALS).

Nutrients	Foods
Vitamin А	Carrots, pumpkin, parsley, peas, broccoli, beef liver, egg yolk, red caviar, butter, milk, cottage cheese, and cheese.
Vitamin В1	Pork, beef, wheat germ and whole grains, organ meats, eggs, fish, legumes, and nuts.
Vitamin В2	Chicken eggs, milk, cottage cheese, cheese, and liver.
Vitamin В6	Pistachios, marjoram, beans, sea buckthorn, salmon, tuna, mackerel, walnuts, liver, hazelnuts, sardines, horseradish, garlic, chili peppers, sweet peppers, millet, chicken, pomegranate, and pine nuts.
Vitamin В7 (В8 and Н)	Meat products, egg yolk, yeast, nuts and seeds, salmon, dairy products, avocados, sweet potatoes, and cauliflower.
Vitamin В9	Liver (chicken, beef, and pork), peanuts; sunflower seeds, lentils, orange juice, parsley, raw beans, avocado, walnuts, spinach, beets; hazelnuts, peas, broccoli, cauliflower, almonds, porcini mushrooms, wild garlic, papaya, and strawberry.
Vitamin В12	Liver (beef, pork, and chicken), octopus, mackerel, sardine, rabbit, beef, sea bass, pork, lamb, cod, carp, Dutch cheese, crab, chicken egg, and sour cream.
Vitamin С	Rowan, strawberry, orange, radish, black currant, apple, lemon, sea buckthorn, cherry, shea, tomato, cabbage, and potatoes.
Vitamin D	Salmon species, herring, cod liver, egg yolk, and cow’s milk.
Vitamin Е	Almonds, hazelnuts, peanuts, pistachios, cashews, dried apricots, sea buckthorn, eel, rose hips, wheat, walnuts, spinach, squid, viburnum, sorrel, salmon, pike perch, prunes, oatmeal, and barley groats.
LDL cholesterol	Red meat, sausages, hard cheeses, bacon, flour confectionery, cream, and hydrogenated vegetable fats.
HDL cholesterol	Olive oil, flaxseed oil, fish oil, nuts, and whole grain wheat products.
PUFAs	Fish oil, sunflower oil, wheat germ oil, peanut oil, soybean oil, olive oil, red caviar, fresh salmon, fresh herring, mackerel, chicken eggs, flax seeds, pine nuts, walnuts, and sprouted wheat grains.

**Table 3:** Recommended average daily intake of nutrients that can be used as promising protectors against ALS.

Nutrients	Daily Requirement in Adults
Vitamin А	1.0 μ
Vitamin В 1	1.6–1.9 μ
Vitamin В2	2.5–3.5 μ
Vitamin В6	2.0 μ
Vitamin В7	150—200 μ
Vitamin В9	200 μ
Vitamin В12	3 μ
Vitamin С	50–60 mg
Vitamin D	1000–2000 IU
Vitamin Е	12–15 IU
PUFAs	Omega-3—1–3 g Omega-6—5–9 g Omega-9—20–30% of daily ration
HDL cholesterol	300 mg
